# Advanced treatment of saline municipal wastewater by *Ruppia maritima*: A data set

**DOI:** 10.1016/j.dib.2017.06.029

**Published:** 2017-06-22

**Authors:** Mehdi Ahmadi, Hamed Saki, Afshin Takdastan, Mehri Dinarvand, Sahand Jorfi, Bahman Ramavandi

**Affiliations:** a Environmental Technologies Research Center, Ahvaz Jundishapur University of Medical Sciences, Ahvaz, Iran; b Department of Environmental Health Engineering, Ahvaz Jundishapur University of Medical Sciences, Ahvaz, Iran; c Forests and Rangelands Research Department, Khuzestan Agricultural and Natural Resources Research and Education Center, Agricultural Research Education and Extension Organization (AREEO), Ahvaz, Iran; d Department of Environmental Health Engineering, Bushehr University of Medical Sciences, Bushehr, Iran

**Keywords:** Natural wastewater treatment, *Ruppia maritima*, Saline wastewater, Municipal wastewater

## Abstract

Saline municipal wastewater treatment is a challenging environmental issue in coastal cities, due to the discharge of saline water into the sewers. The present research article focuses on the phytoremediation of high saline municipal wastewater by *Ruppia maritime*, a widespread plant which can be found in saline medium such as traditional fish ponds, estuaries, tidal flats, salt pans, coastal paddy fields, coastal lagoons, marsh pools, and mangrove salt marshes in Khuzestan province, Iran. The experimental data was obtained using a pilot plant constructed in Chobeineh wastewater treatment plant in Ahvaz city, fed by activated sludge effluent in 3 levels of electrical conductivity (EC) (10, 15, 20 ms cm^−1^), during 45 days of the experiment. Chemical oxygen demand (COD), total nitrogen (TN), total phosphorus (TP) and total suspended solids (TSS) were daily monitored in blank and pilot study. The COD removal decreased from 83.26% to 72.39% by increasing the EC level from 10 to 20 ms cm^−1^, respectively. The experimental data will practically be an appropriate source of information for environmental engineers to design a natural treatment scenario for saline wastewater treatment.

**Specifications Table**

**Table:** 

Subject area	*Environmental Engineering*
More specific subject area	*Natural wastewater treatment*
Type of data	*Table, figure*
How data was acquired	*Data collected from phytoremediation of the saline wastewater with 3* EC *levels in 2 pilot studies.*
Data format	*Analyzed*
Experimental factors	*COD, TN, TP, TSS, NH*_*4*_^*+*^*and NO*_*3*_^*-*^*were daily monitored as a function of different electrical conductivity levels.*
Experimental features	*Advanced treatment of saline municipal wastewater by Ruppia maritima*
Data source location	*Ahvaz, Iran, 31°19′13″N 48°40′09″E*
Data accessibility	*Data are available in the article*

**Value of the data**

• Base on the data set, *Ruppia maritime* is considered as a promising saline tolerant plant in advanced treatment of saline wastewater.
• This data set focused on a challenging issue in treatment of coastal wastewater treatment; therefore, it will be interesting for coastal community with saline wastewaters.
• Our data showed that *R. maritime* could simultaneously remove nutrient and COD from wastewater; an interesting issue for environmentalists who concerned about saline wastewater treatment.

## 1 Data

**Fig. 1:**
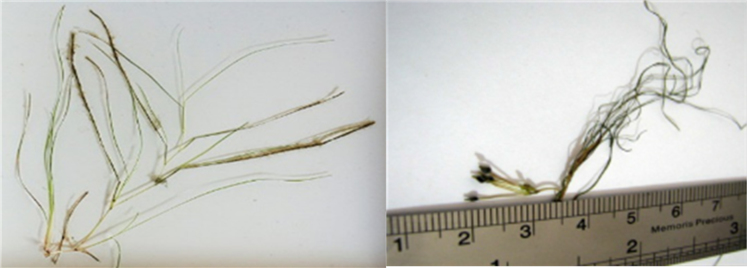
*R. maritime*.

This data set contains 5 tables and 1 figure. Table 1, Table 2, Table 3 represent the performance of *R. maritima* in saline wastewater treatment. Table 4, Table 5 show the specific growth rate and the nutrient uptake rate during the study, respectively. The photo of *R. maritima* plant is presented in Fig. 1.

**Table 1:** Performance of *R. maritima* in saline wastewater treatment (EC = 10 ms cm^−1^).

**Item**	***R. Maritima*****pilot**			**Blank pilot**		
	**Influent (mg L**^**−1**^**)**	**Effluent (mg L**^**−1**^**)**	**Efficiency (%)**	**Influent (mg L**^**−1**^**)**	**Effluent (mg L**^**−1**^**)**	**Efficiency (%)**
**TSS**	45.89 ± 7.38	11.85 ± 2.43	74.17	45.89 ± 7.38	31.08 ± 3.8	32.27
**COD**	61 ± 10.09	10.21 ± 2.12	83.26	61 ± 10.09	49.87 ± 8.38	18.24
**TN**	21.49 ± 2.76	11.65 ± 1.95	45.78	21.49 ± 2.76	19.46 ± 2.5	9.4
**NH**_**4**_^**+**^	6.56 ± 0.92	0.91 ± 0.35	86.12	6.56 ± 0.92	5.93 ± 0.93	9.6
**NO**_**3**_^**−**^	10.67 ± 1.79	4.8 ± 1.37	55.01	10.67 ± 1.79	9.42 ± 1.65	11.7
**TP**	6.41 ± 0.72	1.73 ± 0.37	73.01	6.41 ± 0.72	5.75 ± 0.67	10.3

**Table 2:** Performance of *Ruppia maritima* in saline wastewater treatment (EC = 15 ms cm^−1^).

**Item**	***R. Maritima*****pilot**			**Blank pilot**		
	**Influent (mg L**^**−1**^**)**	**Effluent (mg L**^**−1**^**)**	**Efficiency (%)**	**Influent (mg L**^**−1**^**)**	**Effluent (mg L**^**−1**^**)**	**Efficiency (%)**
**TSS**	41.88 ± 3.29	12.15 ± 3.5	70.98	41.88 ± 3.29	32.28 ± 4.63	22.92
**COD**	55.68 ± 12.45	12.3 ± 2.55	78.08	55.68 ± 12.45	47.04 ± 11.08	15.51
**TN**	21.29 ± 1.44	11.75 ± 0.65	44.8	21.29 ± 1.44	19.39 ± 0.88	8.9
**NH**_**4**_^**+**^	5.48 ± 0.98	0.88 ± 0.13	83.94	5.48 ± 0.98	5.12 ± 0.92	6.5
**NO**_**3**_^**−**^	12.32 ± 1.77	5.42 ± 1.06	56	12.32 ± 1.77	11.19 ± 1.69	9.1
**TP**	5.85 ± 1.13	1.86 ± 0.75	68.2	5.85 ± 1.13	5.32 ± 1.02	9

**Table 3:** Performance of *R. maritima* in saline wastewater treatment (EC = 20 ms cm^−1^).

**Item**	***R. Maritima*****pilot**			**Blank pilot**		
	**Influent (mg L**^**−1**^**)**	**Effluent (mg L**^**−1**^**)**	**Efficiency (%)**	**Influent (mg L**^**−1**^**)**	**Effluent (mg L**^**−1**^**)**	**Efficiency (%)**
**TSS**	45.47 ± 12.88	14.1 ± 3.81	68.99	45.47 ± 12.88	35.35 ± 4.5	22.22
**COD**	56.95 ± 9.98	15.73 ± 2.31	72.39	56.95 ± 9.98	49.65 ± 9.59	12.81
**TN**	22.84 ± 1.77	12.68 ± 1.13	44.48	22.84 ± 1.77	21.2 ± 1.9	7.1
**NH**_**4**_^**+**^	5.52 ± 0.74	0.91 ± 0.12	83.51	5.52 ± 0.74	5.22 ± 1.32	5.4
**NO**_**3**_^**−**^	13.35 ± 3.34	6.27 ± 1.9	53.03	13.35 ± 3.34	12.51 ± 3.4	6.2
**TP**	5.65 ± 0.75	2.07 ± 0.59	63.36	5.65 ± 0.75	5.17 ± 0.71	8.4

**Table 4:** *R. maritima* specific growth rate during the study.

**EC (ms cm**^**−1**^**)**	**Initial dry weight (DW**_**1**_**) (g)**	**Final dry weight (DW**_**2**_**) (g)**	**Specific growth rate (***η***) (g dry wt day**^**−1**^**)**
10	421	757	0.04
15	376	683	0.04
20	310	523	0.03

**Table 5:** *R. maritima* nutrient uptake rate during the study.

**EC (ms cm**^**−1**^**)**	**Nitrogen uptake rate (mg g**^**−1**^**)**	**Phosphorous uptake rate (mg g**^**−1**^**)**
10	0.21	0.029
15	0.18	0.026
20	0.17	0.023

## 2 Experimental design, materials and methods

Secondary effluent of Ahvaz Choneibeh wastewater treatment plant was used as an influent for operation of study and pilot blank.

### 2.1 Pilot plant preparation

Two pilot plants constructed as study and blank pilot (without *R. maritima)*. They were constructed by concrete in a certain dimension (*L* = 3.3 m, *W* = 1.1 m, *H* = 0.8 m). About 7 cm of pilot׳s bed was covered by an appropriate soil layer and prepared for planting. A pump was applied for transition of wastewater from secondary effluent line of the Ahvaz Choneibeh wastewater treatment plant and the flow discharge was adjusted for obtaining the desired detention time (10 days).

In order to prevent the short circuiting flow, two baffles were installed in the entrance and exit of the pilots. In addition, for adjusting the influent EC, a peristaltic pump was used to inject salt solution to influent line to obtain desired EC range as experimental design.

### 2.2 Experimental design

*R. maritima* plant was gathered from a natural wetland around Shosh city, Iran and planted in the prepared pilot study. To determine the specific growth rate and the nutrient uptake rate of *R. maritima*, it was harvested and cleaned from debris and washed by water to separate impurities.

The experiments were conducted with 3 EC ranges (10, 15 and 20 ms cm^−1^) during 45 days in stable situation. For each run the parameters of COD, TSS, NH_4_^+^, NO_3_^−^, TN, TP were daily measured, lack of significance difference in 7 consecutive days base on ANOVA analysis was defined as stable situation [1]. The specific growth rate and the nutrient uptake rate during the study were calculated using Eqs. (1), (2), respectively [2]:

(1) $$\eta =\frac{DW_{2}/DW1}{\Delta T}$$

η= specific growth rate (g dry wt day^−1^)
DW_1_: initial dry weight (g)
DW_2_: final dry weigh (g)
ΔT: duration of study (day)

(2) $$UP=\frac{DW_{2}C_{2}-DW_{1}C_{1}/\Delta T}{\overset{¯}{DW}}$$

UP = uptake rate (mg g^-1^)
$\overset{¯}{DW}=$ Average of initial and final dry weight (g)
C_1_ = initial concentration of TP and TN (mg L^−1^)
C_2_ = final concentration of TP and TN (mg L^−1^)
ΔT = Duration of study (day)

The measurement of COD, TSS, NH_4_^+^, NO_3_^−^, TN, and TP in the influent and effluent of the pilot was done according to methods number of 5220 A, 2540 D, 4500-NH_3_ A, 4500-NO_3_^–^ B, 4500-N C, and 4500-P B, respectively presented in the Standard Methods [1].
